# Clinical Anxiety among Saudi Postgraduate Pediatric Dentistry Students in Jeddah City

**DOI:** 10.1155/2018/5863869

**Published:** 2018-02-27

**Authors:** Manal Almalik, Abeer Alnowaiser, Omar El Meligy, Jamal Sallam, Yusra Balkheyour

**Affiliations:** ^1^Dental Department, King Fahd Armed Forces Hospital, Jeddah, Saudi Arabia; ^2^Pediatric Dentistry Department, Faculty of Dentistry, King Abdulaziz University, Jeddah, Saudi Arabia; ^3^Pediatric Dentistry and Dental Public Health Department, Faculty of Dentistry, Alexandria University, Alexandria, Egypt; ^4^Ministry of Health, Jeddah, Saudi Arabia; ^5^King Abdulaziz University, Jeddah, Saudi Arabia

## Abstract

**Objective:**

To determine anxiety in relation to gender, Grade Point Average (GPA), level of education and academic and clinical situations in Jeddah, Saudi Arabia. Also, to identify academic and clinical anxiety levels among postgraduate pediatric dentistry students.

**Methods:**

A cross-sectional study at governmental training hospitals was conducted. All registered postgraduate students in pediatric dental programs during the year 2015-2016 were included in the study. A self-administered questionnaire was distributed electronically to 60 postgraduate pediatric dentistry students aged between 25 and 45 years old. The questionnaire is composed of 55 questions that investigated demographic data, academic and clinical related situations including investigations, diagnosis, treatment, and complications in treatment.

**Results:**

The study showed a higher anxiety level in younger age dental students (76.7% compared to 23.3%) and Saudi board residents (60%). Comparing gender differences in anxiety revealed that a significant difference (*P* ≤ 0.05) was found and anxiety seems to be more among female dental students (2% very anxious, 64% slightly anxious, and 34% not anxious) as compared to male dental students (8% very anxious, 69% slightly anxious, and 23% not anxious).

**Conclusions:**

There was increased awareness, detailed understanding, and handling of the patients by senior postgraduate pediatric dentistry students compared to junior students.

## 1. Introduction

Anxiety is a psychological and physiological state composed of feeling of worry, nervousness, or unease about something with an uncertain outcome [1].

Anxiety is potentially problematic for both patients and dentists. The origins of dentist anxiety in the dental clinic have a complex and multifactorial psychological and physiological etiology. Among dental students and practitioners, multiple researches were conducted to study the level of anxiety, stress, and their contributing factors [2–4].

The physical symptoms of anxiety may include one or more of the following: feelings of apprehension, trouble concentrating, anticipating the worst, irritability, restlessness, shortness of breath, heart palpitations, dry mouth, nausea, muscle tension, and cold or sweaty hands and feet as well as of numbness or tingling in the hands or feet. On the other hand, the psychological symptoms may also include one or more of the following: problems with concentration, difficulty with staying on task, memory difficulties, depressive symptoms like hopelessness, lethargy, and poor appetite, as well as becoming overly attached to a safety object or person, and finally avoiding crowded places [5–7].

An interesting finding in a cross-sectional study in 2009 by Al-Omari WM and Al-Omiri MK in Jordan on 600 medical, dental, and engineering undergraduate students showed that dental students had the lowest percentage of anxiety [8].

Another study was carried out in 2010 on 815 medical students in Nishtar Medical College and concluded that anxiety and depression were decreased with increasing student's age and with high anxiety levels in female students as compared to male students [9].

Furthermore, a study in 2011 in Plovdiv University, Bulgaria, revealed that dental students have a significantly higher level of dental anxiety at the beginning of their training than at its end [10].

Regarding postgraduate students, a study done in India on 50 postgraduate medical students showed that several factors such as age and type of the course contributed to raise the amount of stress and anxiety among these students [11].

Several studies were carried out to determine the anxiety provoking situations in medical and dental students [12–14]. To our knowledge, there were very few studies available that have investigated the association of anxiety among postgraduate pediatric dentistry students.

The objectives of this study were to determine anxiety in relation to gender, Grade Point Average (GPA), level of education and academic and clinical situations in the city of Jeddah, Saudi Arabia, and also to identify academic and clinical anxiety levels among postgraduate pediatric dentistry students.

## 2. Methods

This cross-sectional study was performed at governmental training hospitals in Jeddah, Kingdom of Saudi Arabia, and data were collected during the year 2015-2016.

Sixty postgraduate pediatric dentistry students (13 males and 47 females) aged between 25 and 45 years old, working in Saudi governmental training hospitals in Jeddah city, were included in the study. Thirty-six were Saudi board residents, 18 were master degree (MSc) students, and 6 were PhD students.

A structured questionnaire was used to gather the participants' data. The questionnaire was reviewed by three experts to ensure that the items were useful; there were agreements between the parts of the questionnaire, and the questions were relevant to the study. The experts rated each question, and their scores were analyzed to calculate the questionnaire's validity.

The self-administered questionnaire was distributed electronically to 60 postgraduate pediatric dentistry students. The questionnaire is composed of fifty-five questions that investigated demographic data, academic, and clinical-related situations including investigations, diagnosis, treatment, and complications in treatment.

Sixty respondents have completed the questionnaire successfully. Uncompleted questionnaires were excluded from the study.

### 2.1. Reliability Test

To determine the questionnaire reliability (intraexaminer), a test was done by distributing the questionnaire to 20 postgraduate pediatric dentistry students working at Saudi governmental hospitals. After 2 weeks, a retest was done by distributing the same questionnaire to the same 20 postgraduate pediatric dentistry students and comparing their responses (test-retest reliability).

### 2.2. Statistical Analysis

Data were fed to the computer using IBM Statistical Package for the Social Sciences (SPSS) software (version 20.0, SPSS Inc., Chicago, IL). The 0.05 level was used to indicate statistical significance.

### 2.3. Ethical Considerations

This study proposal has been approved by the Saudi governmental training hospitals, Jeddah, Saudi Arabia, and has therefore been performed in accordance with the ethical standards laid down in the 1964 Declaration of Helsinki and its later amendments (proposal number 096-01-17).

## 3. Results

Intraexaminer reliability was determined and was 0.95, representing excellent agreement. The overall response rate was 100%. All the 60 (13 males (21.7%) and 47 females (78.3%)) respondents have successfully completed the questionnaire. All the participants were postgraduate pediatric dentistry students aged between 25 and 45 years old. The study showed a higher anxiety level in younger age dental students (76.7% compared to 23.3%), females more than males (78.3% compared to 21.7%). For GPA, 3.3% of the respondents graduated with a GPA of 2.5–3; 76.6% graduated with a GPA of 3.1–4.4, while 20.1% had a GPA of 4.5–5. Regarding current study, 60% (36) of our respondents were Saudi board residents, 30% (18) were MSc students, and 10% (6) were PhD students. Seventeen (28.3%) students were first-year postgraduate dental students, 18 (30%) were second year, and 15 (25%) were third year, while 10 (16.7%) were final-year dental students (Table 1).

**Figure 1:**
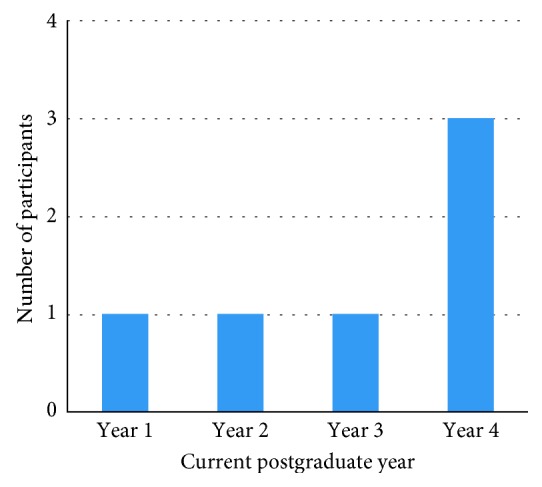
Level of anxiety during clinical investigations with respect to postgraduate current year.

**Figure 2:**
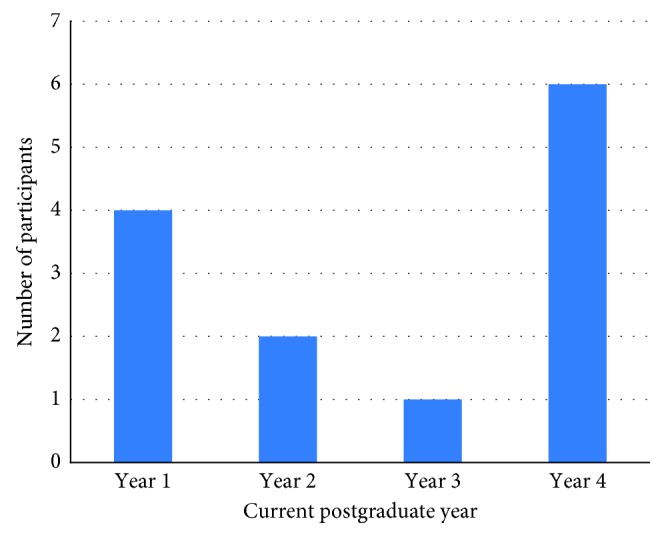
Level of anxiety during clinical diagnosis with respect to postgraduate current year.

The level of clinical diagnosis, investigations, and treatment of the patients with respect to the postgraduate level and gender was the highest anxiety provoking situations for both male and female dental students. The senior postgraduate students in their final year were more likely to be anxious during clinical diagnosis and investigations than the first-year (junior) postgraduate students. There was a statistically significant difference (*P* ≤ 0.05) between first-year and final-year students for the following questions: unable to diagnose patients, misdiagnosing patients, unable to answer the parents' questions as well as taking history and conducting patient examination, poor radiographic interpretation, prescribing medications and writing prescriptions, writing referral forms, referring patients for GA, or conscious sedation when needed (Figures 1 and 2).

**Figure 3:**
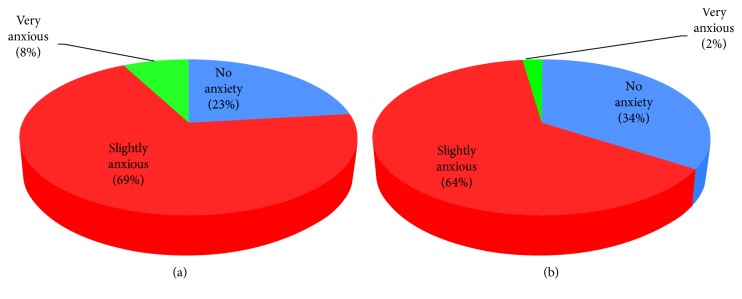
Level of total anxiety in relation to gender. (a) Percentage of total anxiety within males. (b) Percentage of total anxiety within females.

Comparing gender differences in anxiety revealed that a significant difference (*P* ≤ 0.05) was found, and anxiety seems to be more among female dental students (2% very anxious, 64% slightly anxious, and 34% not anxious) as compared to male dental students (8% very anxious, 69% slightly anxious, and 23% not anxious) (Figure 3).

**Figure 4:**
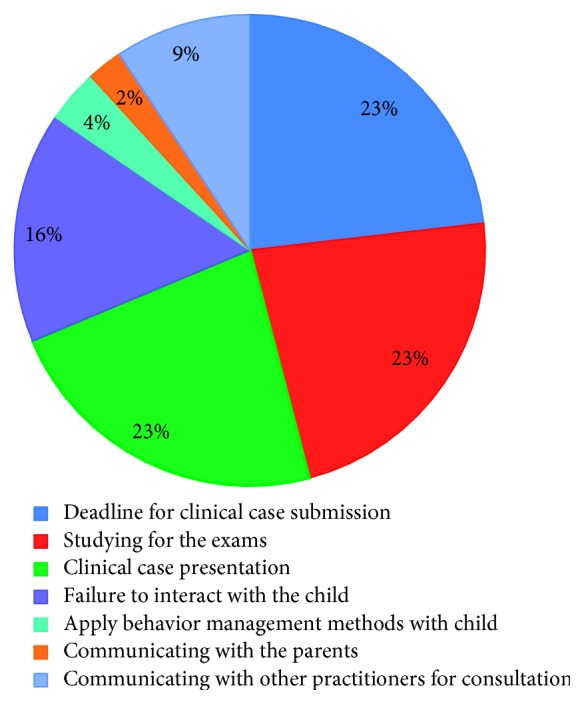
Level of anxiety during academic situations.

Regarding the level of anxiety during academic situations, 23% were anxious regarding deadline for clinical case submission, 23% were anxious regarding studying for their exams, 23% were anxious regarding clinical case presentation, 16% were anxious regarding failure to interact with the child, 4% were anxious regarding application of behavior management methods with the child, 2% were anxious regarding communicating with parents, and 9% were anxious regarding communicating with other practitioners for consultation (Figure 4).

**Figure 5:**
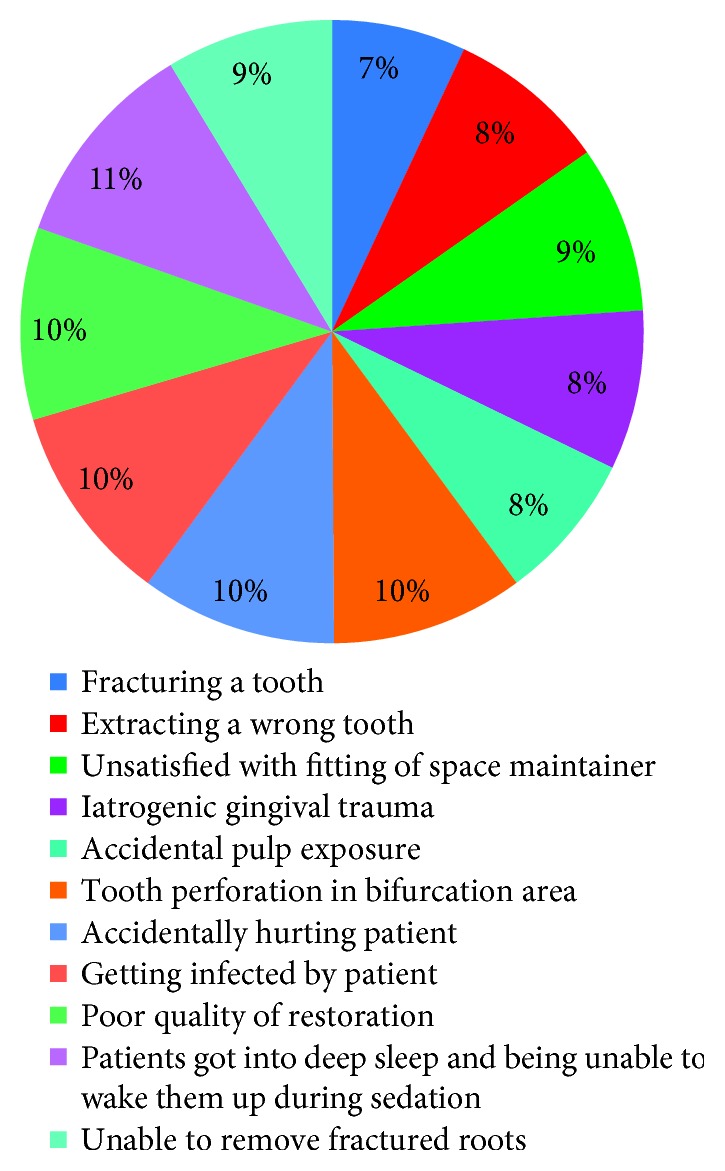
Level of anxiety during clinical complications.

Concerning the level of anxiety during clinical complications, 7% were anxious regarding fracturing a tooth, 8% were anxious regarding extracting the wrong tooth, 9% were anxious being unsatisfied with fitting of space maintainers, 8% were anxious regarding iatrogenic gingival trauma, 8% were anxious regarding accidental pulp exposure, 10% were anxious regarding tooth perforation in bifurcation area, 10% were anxious regarding accidentally injuring the patient, 10% were anxious regarding getting infected by the patient, 10% were anxious regarding poor quality of restorations, 11% were anxious regarding patients got into deep sleep and being unable to wake them up during sedation, and 9% were anxious regarding failure to remove fractured roots (Figure 5).

**Figure 6:**
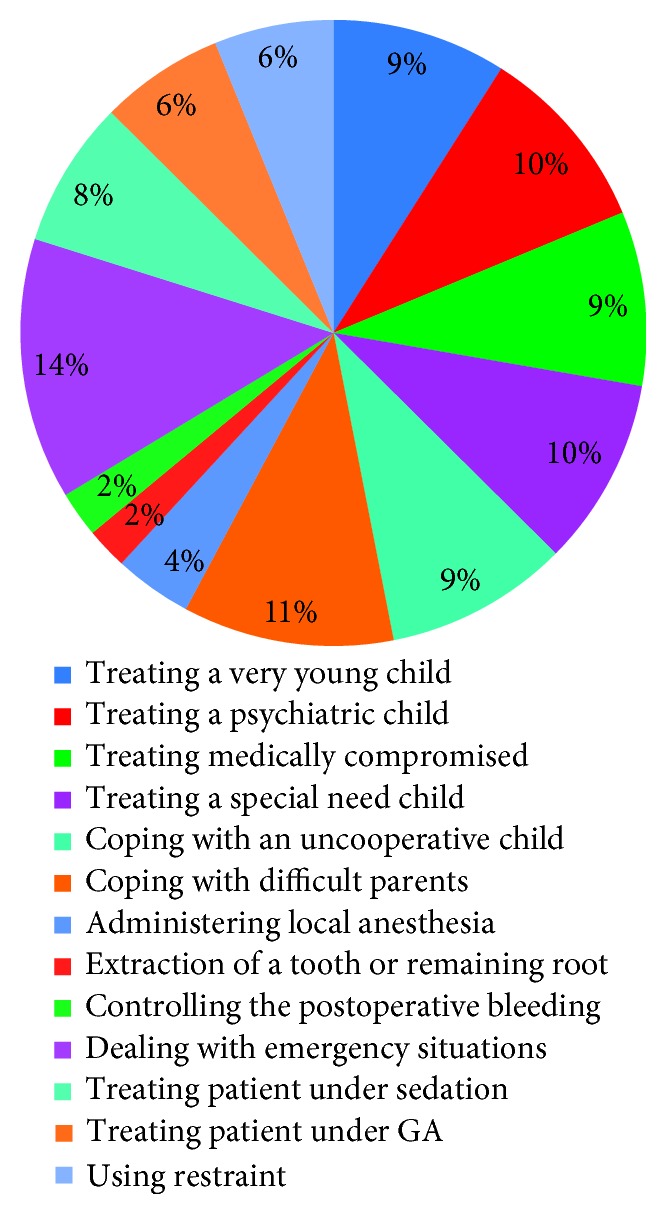
Level of anxiety during various clinical treatments.

With respect to the level of anxiety during various clinical treatments, 9% were anxious when treating a very young child, 10% were anxious when treating psychiatric child, 9% were anxious when treating medically compromised patients, 10% were anxious when treating children with special health care needs, 9% were anxious when coping with uncooperative children, 11% were anxious when coping with difficult parents, 4% were anxious when administering local anesthesia, 2% were anxious during extraction of a tooth or a remaining root, 2% were anxious when controlling postoperative bleeding, 14% were anxious in dealing with emergency situations, 8% were anxious when treating patients under conscious sedation, 6% were anxious when treating patient under general anesthesia (GA), and 6% were anxious using restraints (Figure 6).

## 4. Discussion

Several studies were carried out to determine the anxiety provoking situations in medical and dental undergraduate students. Limited information is available about the association of anxiety among postgraduate pediatric dentistry students. The present study aimed to determine anxiety in relation to gender, GPA, and level of education and academic and clinical situations in the city of Jeddah, Saudi Arabia, and also to identify academic and clinical anxiety levels among postgraduate pediatric dentistry students.

The present study showed a higher anxiety level in younger age dental students (76.7% compared to 23.3%). This is in agreement with a study by Storjord et al. [13], who reported that dental anxiety was less in experienced dental students than new dental students. This strongly suggests that the dental program structure in universities may affect dental anxiety levels.

An explanation for variances in dental anxiety could be that the dental students in the first year are more susceptible to stress and anxiety because they are in an unacquainted study environment. Students just starting their studies can experience more stress due to the challenge of transitioning from high school to university, and a study from the United States showed that seniors and juniors have lower stress reactions than sophomores and freshmen [15]. The decrease in stress due to settling into university life might, therefore, be a generic process leading to lessening of particular anxiety (e.g., dental anxiety). Another explanation is that usually in later years of courses low failure rates make students less stressed and more self-confident [16].

Our study showed a higher anxiety level in females more than males (78.3% compared to 21.7%). In accordance with other studies, female students had reported higher scores of depression, anxiety, and stress compared with their male counterparts [17, 18]. This may be due to the fact that women articulate depressive symptoms, even minor ones, more easily [19]. Also, this dissimilarity may be described by women being better able to show their feelings of fear. Furthermore, physiological conditions such as fear, stress, depression, panic, and social phobia are more prevalent in females, and dental anxiety may be related with such feelings [20].

The current study showed an interesting finding, as senior postgraduate students in their final year were more likely to be anxious during clinical diagnosis and investigations than the first-year (junior) postgraduate students. Anxiety that have been reported during the final year may be due to nonsupportive and extremely perplexing environments as compared to the first year. This is in agreement with a study by Kulsoom and Afsar [21], who reported high stress and anxiety among first-year scholars, which climaxed in the fourth year, where the clerkship phase starts and students rotate through various disciplines of hospital as their chief training method. Also, they observed that the highest anxiety and depression results were reported in the fourth year.

Our study is in accordance with that of Alpert and Haber [22], where the level of anxiety during academic situations was the highest (23%) regarding deadline for clinical case submission, studying for their exams and clinical case presentation, while only 2% were anxious regarding communicating with parents.

Concerning the level of anxiety during clinical complications, the highest (11%) were anxious regarding patients got into deep sleep and being unable to wake them up during sedation, while the lowest (7%) were anxious regarding fracturing a tooth. This is in agreement with a study by Basudan et al. [14], who reported high occurrence of stress, anxiety, and depression among dental students.

Regarding the level of anxiety during various clinical treatments, the highest (14%) were anxious in dealing with emergency situations; however, 2% were anxious during extraction of a tooth or a remaining root and when controlling postoperative bleeding. This agrees with Ali et al. [23] who concluded that higher anxiety levels were reported by clinical and preclinical students during various clinical procedures.

Our results can point out that the dentistry program structure can be a major cause in decreasing dental anxiety.

The present study has several limitations. First, this study was a cross-sectional observational study using postgraduate pediatric dentistry students from only governmental training hospitals in Jeddah city, which means that findings may indicate institutional characteristics not representative of postgraduate pediatric dentistry students elsewhere in Saudi Arabia. Second, although all postgraduate pediatric dentistry students in governmental training hospitals in Jeddah city were included in this study, still the sample size is considered small to show significant differences in many areas in the study. Third limitation of this study was the lack of funding for the research project.

## 5. Conclusions

The study assists to understand the topic of anxiety among postgraduate dentists. There was increased awareness, detailed understanding, and handling of the patients by senior postgraduate pediatric dentistry students compared to junior students. The high anxiety levels seen in dental students indicate the need to provide support programs and to include stress management courses in their training programs.

## 6. Recommendations

Future research to further understand the topic of anxiety among postgraduate dentists is needed.

## Figures and Tables

**Table 1:** The sociodemographic information of the participants.

Demographic data			
		Number (*N*)	Marginal percentage
Age (years)	25–35	46	76.7
	36–45	14	23.3
Grade Point Average (GPA)	2.5–3	2	3.3
	3.1–4.4	46	76.6
	4.5–5	12	20.1
Gender	Male	13	21.7
	Female	47	78.3
Postgraduate pediatric dentistry students	Saudi board	36	60
	MSc	18	30
	PhD	6	10
Current postgraduate year	1	17	28.3
	2	18	30
	3	15	25
	4	10	16.7
Total		60	100
